# IRF2 regulates cellular survival and Lenvatinib-sensitivity of hepatocellular carcinoma (HCC) through regulating β-catenin

**DOI:** 10.1016/j.tranon.2021.101059

**Published:** 2021-03-15

**Authors:** Yarong Guo, Jun Xu, Qiang Du, Yihe Yan, David A. Geller

**Affiliations:** a Thomas E. Starzl Transplantation Institute, Department of Surgery, University of Pittsburgh Medical Center, Pittsburgh, PA 15260, United States; b Department of Oncology, The First Affiliated Hospital of Shanxi Medical University, Taiyuan 030001, Shanxi, China; c Department of Surgery, The First Affiliated Hospital of Shanxi Medical University, Taiyuan 030001, Shanxi, China

**Keywords:** Hepatocellular carcinoma, Interferon regulatory factor 2 (IRF2), β-catenin, Cellular apoptosis, Lenvatinib-resistance

## Abstract

Hepatocellular carcinoma (HCC) is the third leading cause of cancer-related death worldwide. Lenvatinib oral chemotherapy is approved as a first-line treatment of patients with unresectable HCC. The efficacy and therapeutic duration of lenvatinib are limited by drug resistance, and the mechanism is unclear. IRF2 is a constitutive transcription factor associated with the development of various cancers by regulating cancer cell growth, apoptosis, and drug resistance. However, the potential role of IRF2 in lenvatinib resistance in HCC has not been explored. In this study, we found that IRF2 promoted proliferation, inhibited apoptosis, and increased lenvatinib resistance of HCC cells by regulating β-catenin expression. Silencing IRF2 downregulated the expression of β-catenin, while overexpressing IRF2 upregulated β-catenin. Moreover, the expression of β-catenin and IRF2 was positively correlated in HCC tissues. Inhibiting β-catenin with XAV-939 effectively abrogated β-catenin expression caused by lenvatinib treatment. These findings identify an important function of IRF2 in HCC and demonstrate a mechanism of lenvatinib resistance of HCC cells. Targeting IRF2 may be a potential strategy to improve the therapeutic effect of lenvatinib on HCC.

## Highlights

• IRF2 and β-catenin are highly expressed in HCC tissues.
• IRF2 upregulation of β-catenin promotes HCC cell proliferation.
• IRF2 enhances lenvatinib resistance in HCC cells.

## Introduction

Hepatocellular carcinoma (HCC) is the third leading cause of cancer-related death worldwide, with a high incidence of HCC in the African and Asian-Pacific regions [1]. Hepatitis C virus (HCV), hepatitis B virus (HBV) infection, heavy alcohol intake and nonalcoholic fatty liver disease are risk factors for HCC [2]. Surgical resection, local ablation, chemotherapy, molecular targeted therapy and liver transplantation are major treatment options for HCC [3,4]. However, the majority of patients with HCC are unresectable and the prognosis remains poor.

Lenvatinib, an oral multi-kinase inhibitor, is recommended as the first-line treatment of patients with unresectable HCC in the USA, EU, Japan, and China since 2019. Lenvatinib shows non-inferiority for overall survival (OS), but significantly improves the objective response rate (ORR) and progression-free survival (PFS) [5]. Importantly, lenvatinib exhibited a favorable tolerability profile compared with sorafenib [5]. Nevertheless, the efficacy and therapeutic duration of lenvatinib are also limited by acquired or intrinsic resistance [6]. Exploring the potential biomarkers and molecular mechanisms of resistance might help to elucidate strategies to improve the therapeutic effect of lenvatinib for HCC.

Interferon regulatory factors (IRFs) are crucial nuclear transcription factors, consisting of 9 members (IRF1–9) in mammals. The IRF family is best known for regulation of gene expression that underlies IFN responses. IRF proteins have a conserved amino-terminal DNA-binding domain (DBD), and recognize a consensus DNA sequence element termed ISRE [7]. IRF proteins play pivotal roles in regulating immune response, lymphocyte differentiation, hematopoietic stem cell development and cell proliferation and tumorigenesis [8]. Among this family, IRF2 is associated with the development of various cancers by regulating gene transcription, such as TP53, CXCL3, Bcl-2, and Bax [9], [10], [11]. IRF2 is upregulated in pancreatic cancer and HCC cells, and high levels of IRF2 are associated with a worse feature of tumor infiltration depth [12,13]. However, the potential role of IRF2 in lenvatinib resistance in HCC has not been characterized.

β-catenin, a subunit of the cell surface cadherin protein complex, is a critical component of Wnt signaling, which is involved in several physiologic and pathophysiologic processes during embryonic development and carcinogenesis. Activation of Wnt signaling induces nuclear accumulation of β-catenin to form a transcription complex and activate the transcription of downstream target genes [14]. Aberrant activation of Wnt/β-catenin signal has been found in several tumor types promoting cancer cell growth, inducing epithelial-to-mesenchymal transition (EMT), conferring stem cell-like features, and developing therapeutic resistance [15], [16], [17]. Likewise, Wnt/β-catenin pathway is frequently activated in HCC, resulting in tumor metastasis, drug resistance and malignant progression [4,18].

In this study, we found that IRF2 was positively correlated with the malignancy of HCC. IRF2 promoted proliferation, inhibited apoptosis, and increased lenvatinib resistance of HCC cells through regulating β-catenin expression. The inhibitor of β-catenin XAV-939 effectively inhibited β-catenin expression caused by lenvatinib treatment. Therefore, our findings suggest that targeting IRF2 may be a potential strategy to improve the therapeutic effect of lenvatinib for HCC.

## Materials and methods

### Cells and specimens

Human HCC cells HepG2 and Huh7 cells were obtained from the American Type Culture Collection and cultured as recommended. The cells were tested negative for mycoplasma contamination and were authenticated by short tandem repeat (STR) fingerprinting. The HCC tissue samples and paired adjacent tissue samples were obtained from University of Pittsburgh Medical Center (UPMC). Written informed consent was obtained from all the patients and participants. This study was approved by the Ethics Committee of University of Pittsburgh Institutional Review Board (IRB).

### Cell viability analysis

CellTiter 96^Ⓡ^ AQueous Non-Radioactive Cell Proliferation Assay (Promega; G5421) was used to detect the viability of HCC cells. 5000 HepG2 and Huh7 cells infected with control or IRF2 overexpression adenoviruses. Control or IRF2-targeting siRNA were plated in 96 well plates. 20μl of the combined MTS/PMS solution was pipetted into each well of the 96 well assay plate containing 100μl of cells in the culture medium. Plates were incubated for 1–4 h at 37 °C in a humidified, 5% CO2 atmosphere. Absorbance was recorded at 490 nm using an ELISA plate reader.

### TUNEL Alexa fluor imaging assay

Click-iT^Ⓡ^ TUNEL Alexa Fluor^Ⓡ^ Imaging Assay (Invitrogen; C10245) was used to detect the apoptotic HCC cells. Briefly, HepG2 and Huh7 cells infected with control or IRF2 overexpressing adenoviruses, or control and IRF2-targeting siRNA were fixed, and cellular permeabilization was conducted. TdT reaction cocktail was added to each sample and incubated for 60 min at 37 °C. Click-iT reaction cocktail was added to each sample and incubated on coverslips for 30 min at room temperature. For DNA staining, the DAPI mounting reagent was used.

### Immunoblotting analysis

25–50 μg of each protein sample was loaded and separated by 10% SDS-PAGE. Proteins were transferred to PVDF membranes, blocked by 5% milk for 0.5–1 h, and incubated with antibodies against Bcl-2 (CST, #3498), survivin (CST, #2808), PARP (CST, #9542), cleaved Caspase-3 (CST, #9661), c-Myc (CST, #13,987), Cyclin-D1 (CST, #2926), IRF2 (CST, #4943), β-catenin (Sigma, C7207), VEGFR2 (CST, #2479), p65 (CST, #8242), Phospho-p65 (Ser536) (CST, #3033) and β-actin (abcam, ab8226) overnight at 4 °C and incubated with secondary antibodies for 2 h at room temperature.

### Immunofluorescence staining analysis

HepG2 and Huh7 cells treated with 0 or 7.5 µM of lenvatinib for 48 h were fixed, blocked by 5% bovine serum albumin (BSA) for 30 min, and incubated with the antibodies against IRF2 or β-catenin at 4 °C overnight. Then the cells were incubated with anti-rabbit or anti-mouse IgG (*H* + *L*). For nuclear staining, DAPI mounting reagent was used.

### RNA isolation and qPCR analysis

The total RNA of HepG2 cells, Huh7 cells, or HCC tissue and paired adjacent tissue samples were isolated with TRIzol reagent and the reverse-transcription was conducted by using Prime Script RT Master Mix Kit (TaKaRa, Tokyo, Japan). Real-time PCR was conducted by using GoTaq qPCR Master Mix (Promega, Madison, WI, USA). Data were analyzed and normalized to the β-Actin data.

### Statistical analysis

The data are shown as the mean ± standard deviation and were analyzed by Student's t-test, a *P*-value < 0.05 was considered to indicate statistical significance. Kaplan–Meier method and the log-rank test were used to analyze the correlation between IRF2 expression and prognosis. Pearson correlation coefficients were used to analyze the expression correlations between IRF2 and β-catenin.

## Results

### IRF2 was increased in HCC and positively correlated with β-catenin expression

**Fig. 1:**
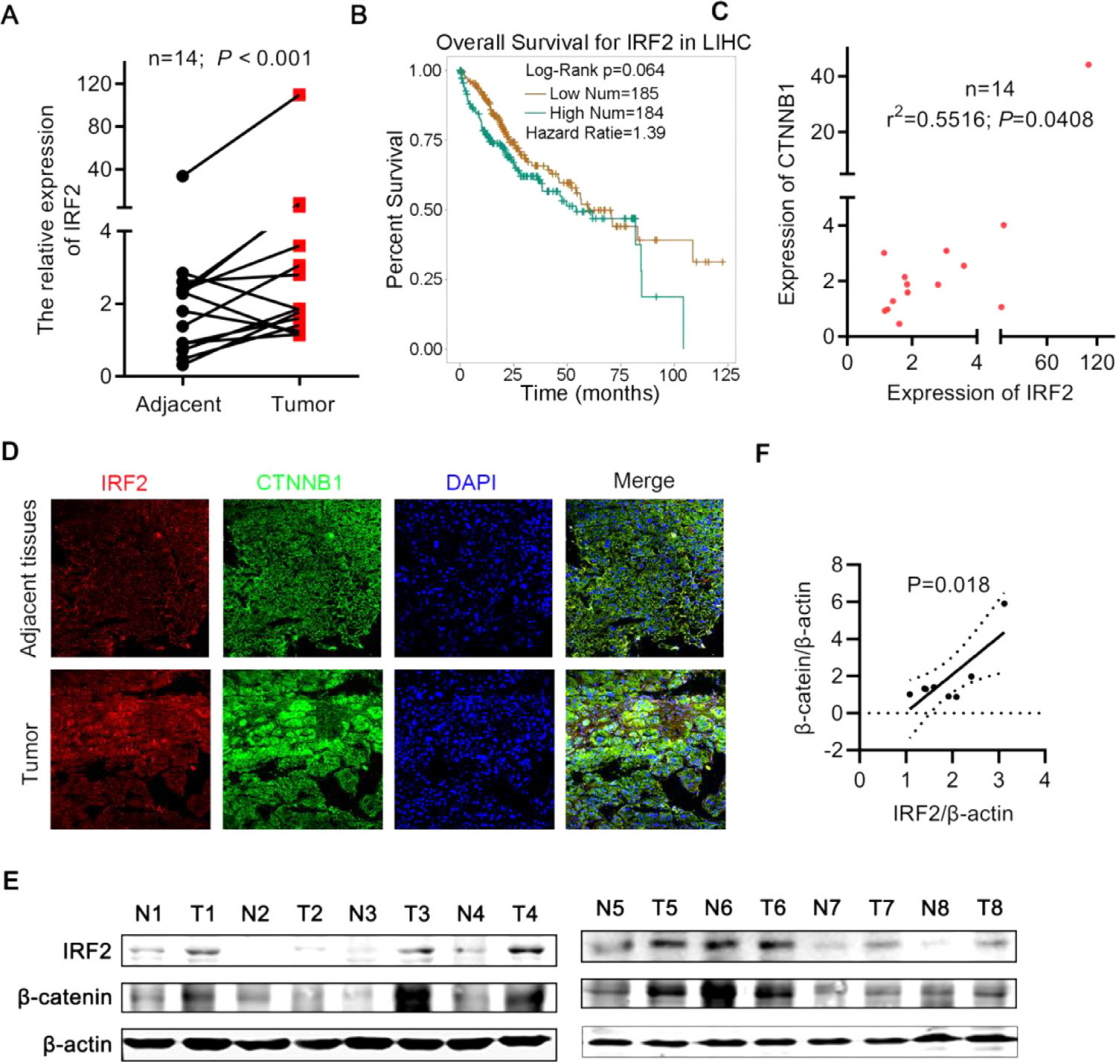
**IRF2 was increased in HCC and positive correlated with β-catenin expression. A.** The relative RNA expression of IRF2 in 14 HCC samples and 14 paired adjacent normal samples from our hospital were detected by real-time PCR. Student's t-test; **B.** The overall survival of patients with low IRF2 expression (*n* = 185) and high IRF2 expression (*n* = 184) in 369 HCC tissue samples. Student's t-test, Kaplan–Meier method and the log-rank test were used; **C.** Correlation between the RNA expression of IRF2 and β-catenin in 14 HCC samples. The Pearson correlation coefficient (r^2^) and *P* value are shown; **D.** Immunofluorescence staining of IRF2 (red), β-catenin (green) and DAPI (blue) in HCC tissues and paired adjacent normal tissues; **E.** Immunoblotting of IRF2 expression and β-catenin expression in 8 HCC samples and 8 paired adjacent normal samples from our hospital. Student's t-test. **F.** Correlation between the protein expression of IRF2 and β-catenin in 8 HCC samples as described in Fig. 1F.

To address the correlation between the expression of IRF2 and the malignancy of HCC. We analyzed the expression of IRF2 in 14 HCC tumors and paired adjacent tissues. IRF2 was dramatically increased in HCC compared to adjacent non-cancer liver tissues (Fig. 1A). To verify the correlation between IRF2 expression and prognosis of HCC, 369 HCC tissue samples from TCGA database were divided into low and high IRF2 groups. Patients with high IRF2 expression exhibited a trend towards shorter overall survival times (Fig. 1B).

Since β-catenin mutation occurs in many HCC tumors, we examined the relationship between β-catenin expression and IRF2. Interestingly, we found a positive correlation between IRF2 and β-catenin expression (Fig. 1C). To examine the expression of IRF2 and β-catenin protein, we performed immunofluorescence and western blot with 8 HCC tumors and paired adjacent tissues. IRF2 expression was increased in 7 of 8 HCC tumors compared to background liver, while β-catenin was increased in 5 of 8 tumors (Figs. 1D and 1E). The expression of IRF2 and β-catenin was positively correlated (Fig. 1F).

Since IRF2 and β-catenin were co-induced in many HCC tumors, this raised the possibility that β-catenin may be a target gene of IRF2, and that IRF2 affects the growth of HCC cells through β-catenin.

### IRF2 induces β-catenin, promotes cellular proliferation, and inhibits apoptosis in HCC

**Fig. 2:**
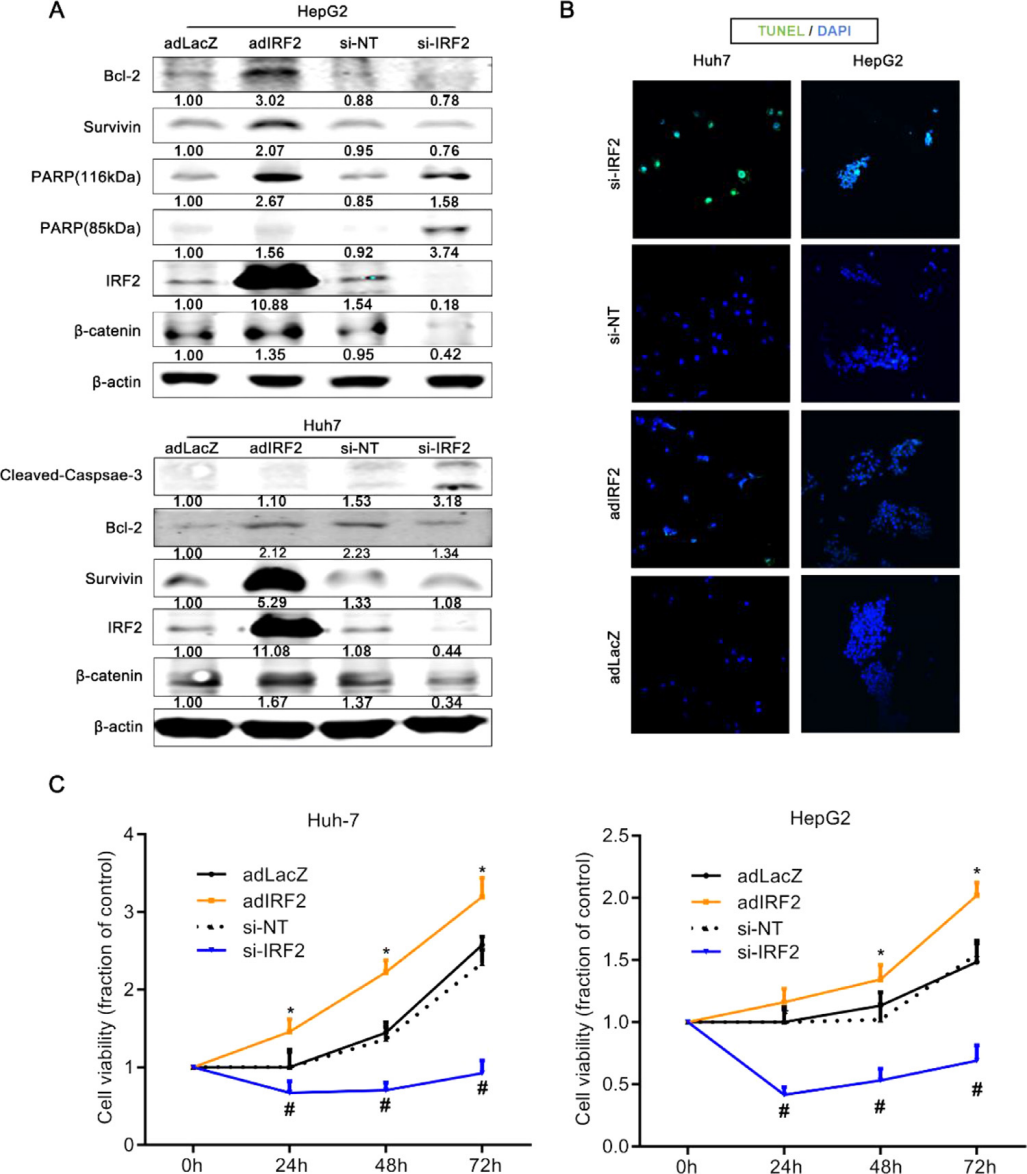
**IRF2 promotes cellular proliferation and inhibits cellular apoptosis in HCC. A.** Immunoblotting of Bcl-2, survivin, 89 kD PARP, 116 kD PARP, IRF2 and β-catenin in HepG2 cells infected with negative control and IRF2 overexpression adenoviruses or control and IRF2-targeting siRNA (top panel). Immunoblotting of cleaved Caspase-3, Bcl-2, survivin, IRF2 and β-catenin in Huh7 cells infected with negative control and IRF2 overexpression adenoviruses or control and IRF2-targeting siRNA (bottom panel). β-actin was used as loading control. The quantified levels of these proteins were described below; **B.** Immunofluorescence staining of TUNEL (green) and DAPI (blue) in HepG2 and Huh7 cells infected with negative control and IRF2 overexpression adenoviruses, or HepG2 and Huh7 cells transfected with control and IRF2-targeting siRNA; **C.** Cell viability of HepG2 and Huh7 cells infected with negative control and IRF2 overexpression adenoviruses, or HepG2 and Huh7 cells transfected with control and IRF2-targeting siRNA. Student's t-test.

Wnt/β-catenin signaling regulates cellular apoptosis in many cancer cells. To examine for a relationship between IRF2 and β-catenin, we overexpressed IRF2 or silenced endogenous IRF2 in human hepatoma HepG2 and Huh7 cell lines. Overexpressing IRF2 with AdIRF2 transduction increased the expression of Bcl-2, survivin and _116kDa_PARP, which inhibit cellular apoptosis, and promoted the expression of β-catenin in HCC cells (Fig. 2A). AdLacZ transduction was used as control.

Knockdown of IRF2 protein with si-IRF2 (but not si-NT negative control) decreased the expression of endogenous IRF2, and likewise decreased the expression of β-catenin in HCC cells (Fig. 2A). Interestingly, the silencing of constitutive IRF2 with si-IRF2 increased cleaved caspase-3 (Fig. 2A, bottom panel).

Next, to examine the functional effect on apoptosis of the HCC cells, TUNEL staining was performed. Knockdown of IRF2 increased the percentage of apoptotic cells in HepG2 and Huh7 cells (Fig. 2B). Furthermore, cellular proliferation of HepG2 and Huh7 cells was significantly promoted when IRF2 was overexpressed, while cellular proliferation was markedly inhibited with knockdown of endogenous IRF2 (Fig. 2C). These data suggest that IRF2 facilitates cellular proliferation and suppresses cellular apoptosis of HCC cells, indicating an important role in regulating HCC survival. This was mediated in part by the upregulation of β-catenin and several apoptosis proteins.

### IRF2 and β-catenin are upregulated in lenvatinib-treated HCC cells

**Fig. 3:**
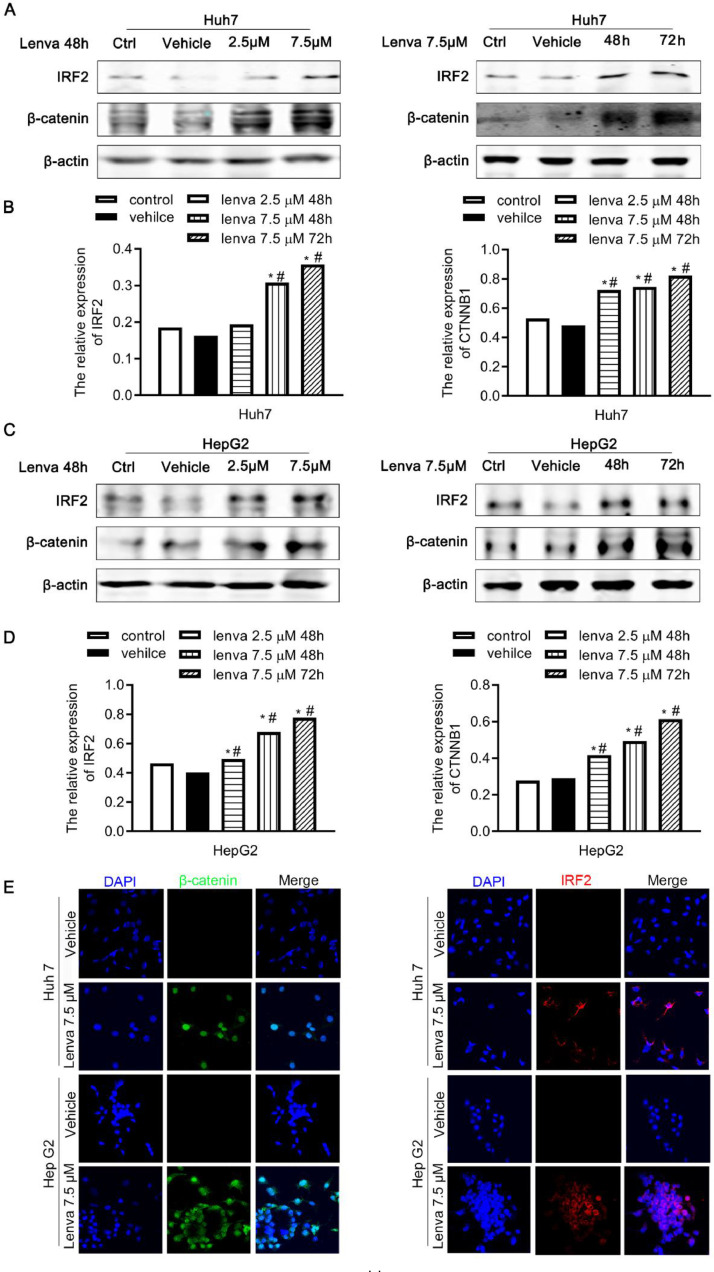
**IRF2 and β-catenin are upregulated in lenvatinib treated HCC cells. A.** Immunoblotting of IRF2 and β-catenin in Huh7 cells treated with 0, 2.5, 7.5 µM of lenvatinib for 48 h (left panel). And immunoblotting of IRF2 and β-catenin in Huh7 cells treated with 0, 7.5 µM of lenvatinib for 48 h or 72 h (right panel); **B.** mRNA levels of IRF2 and β-catenin in Huh7 cells treated with 0, 2.5, 7.5 µM of lenvatinib for 48 h (left panel). And mRNA levels of IRF2 and β-catenin in Huh7 cells treated with 0, 7.5 µM of lenvatinib for 48 h or 72 h (right panel). Student's t-test; **C.** Immunoblotting of IRF2 and β-catenin in HepG2 cells treated with 0, 2.5, 7.5 µM of lenvatinib for 48 h (left panel). And immunoblotting of IRF2 and β-catenin in HepG2 cells treated with 0, 7.5 µM of lenvatinib for 48 h or 72 h (right panel); **D.** mRNA levels of IRF2 and β-catenin in HepG2 cells treated with 0, 2.5, 7.5 µM of lenvatinib for 48 h (left panel). And mRNA levels of IRF2 and β-catenin in HepG2 cells treated with 0, 7.5 µM of lenvatinib for 48 h or 72 h (right panel). Student's t-test; **E.** Immunofluorescence staining of IRF2 (red), β-catenin (green) and DAPI (blue) in HepG2 and Huh7 cells treated with 0 and 7.5 µM of lenvatinib for 48 h.

To determine the effect of lenvatinib treatment on IRF2 and β-catenin expression in HCC cells, HepG2 and Huh7 cells were treated with 0, 2.5, or 7.5 µM of lenvatinib for 48 h or 72 h. Surprisingly, lenvatinib increased the expression of IRF2 and β-catenin in HepG2 and Huh7 cells in a dose- and time-dependent manner (Fig. 3A – 3D). We further confirmed this result by using immunofluorescence staining, which showed that lenvatinib (7.5 µM) increased the expression of IRF2 and β-catenin proteins *in situ* (Fig. 3E). These results suggest a possible defense mechanism by HCC cancer cells where induction of IRF2 and β-catenin in response to lenvatinib occurs to resist lenvatinib mediated cellular apoptosis, thereby promoting HCC survival

### IRF2 overexpression decreases the Lenvatinib sensitivity of HCC cells

**Fig. 4:**
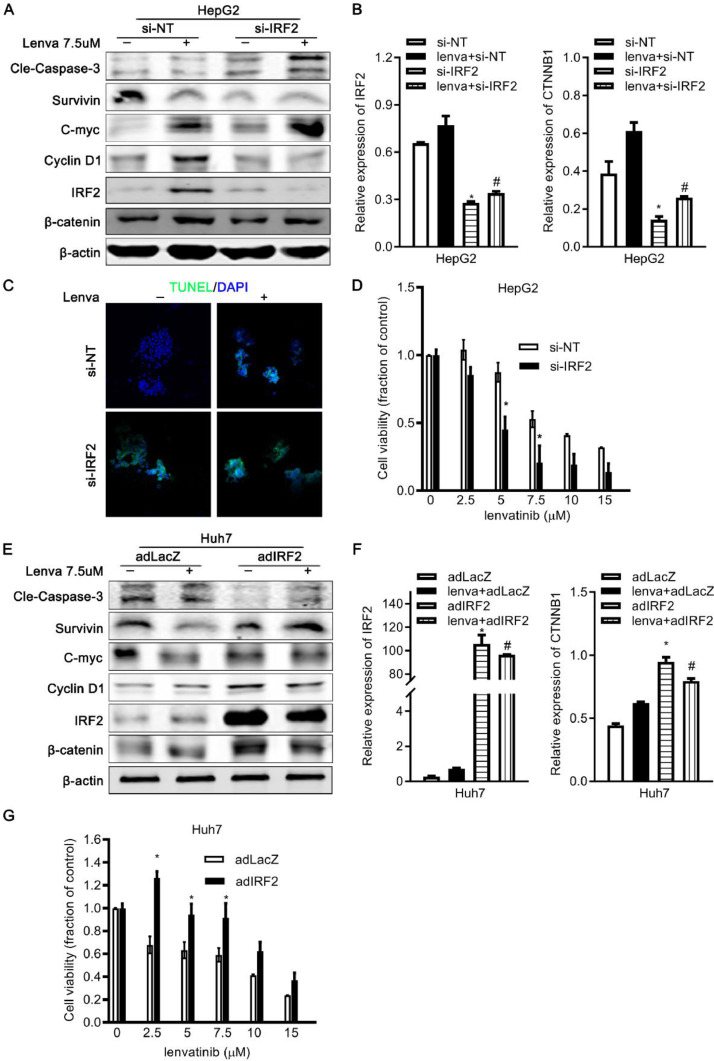
**IRF2 overexpression decreases lenvatinib sensitivity and cellular apoptosis of HCC cells. A.** Immunoblotting of cleaved Caspase-3, survivin, C-myc, Cyclin-D1, IRF2 and β-catenin in control and IRF2-knockdown HepG2 cells treated with 7.5 µM of lenvatinib for 48 h. β-actin was used as loading control; **B.** mRNA levels of IRF2 and β-catenin in control and IRF2-knockdown HepG2 cells treated with 7.5 µM of lenvatinib for 48 h as described in Fig. 4a. β-actin was used as loading control. Student's t-test; **C.** Immunofluorescence staining of TUNEL (green) and DAPI (blue) in control and IRF2-knockdown HepG2 cells treated with 7.5 µM of lenvatinib for 48 h; **D.** Cell viability of control and IRF2-knockdown HepG2 cells treated with 0, 2.5, 5, 7.5, 10, 15 µM of lenvatinib for 48 h. Student's t-test; **E.** Immunoblotting of cleaved Caspase-3, survivin, C-myc, Cyclin-D1, IRF2 and β-catenin in control and IRF2-overexpressing Huh7 cells treated with 7.5 µM of lenvatinib for 48 h. β-actin was used as loading control; **F.** mRNA levels of IRF2 and β-catenin in control and IRF2-overexpressing Huh7 cells treated with 7.5 µM of lenvatinib for 48 h as described in Fig. 4e. β-actin was used as loading control. Student's t-test; **G.** Cell viability of control and IRF2-overexpressing Huh7 cells treated with 0, 2.5, 5, 7.5,10, 15 µM of lenvatinib for 48 h. Student's t-test.

To further explore the relationship between IRF2 expression, β-catenin, and lenvatinib resistance of HCC, we overexpressed or silenced endogenous IRF2 in HCC cells. The expression of apoptosis-related genes was analyzed in control and IRF2-knockdown HepG2 cells treated with or without 7.5 µM of lenvatinib for 48 h. With lenvatinib treatment, the silencing of endogenous IRF2 increased the expression of cleaved Caspase-3 and decreased the expression of survivin and β-catenin (Fig. 4A, 4B).

C-Myc and Cyclin D1 are target genes of Wnt/β-catenin signaling pathway and play a role in cell cycle progression, apoptosis, and may also regulate the sensitivity of cancer cells to chemotherapy. The expression of c-Myc was increased and the expression of Cyclin D1 was decreased in control and IRF2-knockdown HepG2 cells treated with Lenvatinib (Fig. 4A, 4B).

TUNEL assay showed that apoptotic cells increased in IRF2-knockdown HepG2 cells treated with Lenvatinib (Fig. 4C). Cellular proliferation in HepG2 cells was decreased by Lenvatinib in a dose-dependent manner, while knockdown of IRF2 augmented Lenvatinib sensitivity resulting in greater cell viability at all doses tested (Fig. 4D). Likewise, cellular apoptosis proteins were reduced and cellular proliferation was increased in IRF2-overexpressing Huh7 cells treated with Lenvatinib (Figs. 4E, 4F). In contrast, overexpression of endogenous IRF2 decreased Lenvatinib sensitivity and enhanced cell viability (Fig. 4G).

### XAV-939 inhibits β-catenin signaling and induces cellular apoptosis in HCC cells with Lenvatinib treatment

**Fig. 5:**
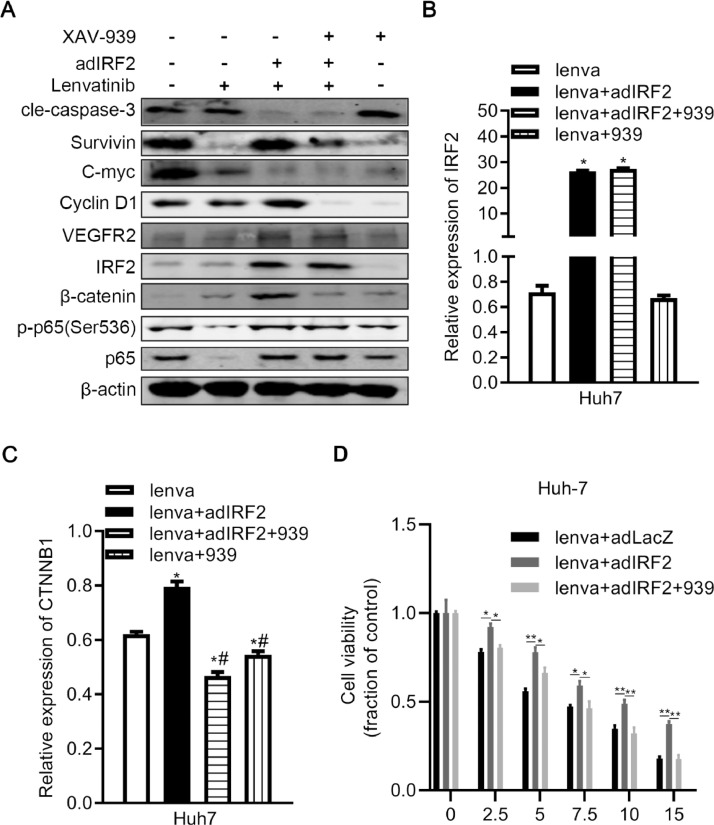
**XAV-939 inhibits β-catenin signal and induces cellular apoptosis in HCC cells with lenvatinib treatment. A.** Immunoblotting of Caspase-3, survivin, C-myc, Cyclin-D1, IRF2, phosphorylated p65, p65, VEGFR2 and β-catenin in control and IRF2-overexpressing lenvatinib-treated Huh7 cells treated with or without the inhibitor of β-catenin XAV-939. β-actin was used as internal control; **B-C.** qPCR of IRF2 (B) and β-catenin (C) in control and IRF2-overexpressing lenvatinib-treated Huh7 cells treated with or without the inhibitor of β-catenin XAV-939. Student's t-test. **D.** Cell viability of control and IRF2-overexpressing lenvatinib-treated Huh7 cells treated with or without the inhibitor of β-catenin XAV-939. Cells were treated with 0, 2.5, 5, 7.5,10, 15 µM of lenvatinib for 48 h. Student's t-test.

β-catenin affects the transcription of c-Myc and Cyclin D1, thereby regulating cellular apoptosis, proliferation, and Lenvatinib resistance of cancer cells. We found that IRF2 may regulate the cellular apoptosis, proliferation and Lenvatinib resistance through β-catenin. XAV-939 is a small molecule and selective Wnt pathway β-catenin-mediated transcription inhibitor. We used XAV-939 and Lenvatinib to treat control or IRF2-overexpressing Huh7 cells. Treatment of XAV-939 did not affect the expression of IRF2 (Figs. 5A and 5B). XAV-939 reduced the expression of β-catenin in Huh7 cells treated with Lenvatinib, and also reversed the upregulated β-catenin expression induced by overexpressing IRF2 (Figs. 5A and 5C). XAV-939 also increased the expression of cleaved caspase-3 and decreased the expression of survivin and phosphorylated p65 in IRF2-overexpressing cells treated with Lenvatinib compared to the control group without XAV-939 treatment (Fig. 5A). Moreover, the expression of c-Myc and Cyclin D1 (target genes of β-catenin) are decreased and cellular viability was suppressed when treated with XAV-939 in IRF2-overexpressing cells treated with Lenvatinib (Figs. 5A and 5D). In summary, a combination of Lenvatinib and XAV-939 treatment effectively decreased the expression of β-catenin, and the reduction of β-catenin may impair cellular survival and Lenvatinib resistance of HCC cells.

## Discussion

IRF2 is reported to influence the occurrence and development of cancer via altering its target genes involved in cell proliferation, apoptosis, and metastasis. Endogenous IRF2 depletion induced testicular embryonal carcinoma (NT2) cell apoptosis and impaired proliferation through promoting the expression of TP53 [11]. The overexpression of IRF2 increased the expressions of cyclin D1, consequently reduced apoptosis and contributed to cell growth in pancreatic cancer [13]. *In vivo* studies showed that the overexpression of IRF-2 resulted in larger tumors than control in ESCC and melanoma mouse model [30,31]. Further, when the IRF-2 gene was overexpressed in NIH 3T3 cells, the cells transformed and displayed enhanced tumorigenicity in nude mice [32]. Moreover, Fas-associated phosphatase 1 (Fap1) gene promoter, a ubiquitously expressed tyrosine phosphatase, was repressed by IRF2, impairing the stem cell-like features in a murine xenograft model of colon cancer [19]. However, some studies showed that IRF2 plays a tumor suppressor role in HBV or HCV associated HCC, and IRF2 silencing significantly increased cell proliferation by impairing the function of TP53 [33], which may be caused by the heterogeneity of tumor cells. Hence, IRF2 may regulate different signaling pathways to either promote or suppress the development of cancer. Here, we showed that IRF2 increases the expression of β-catenin and regulates Cyclin D1. Since Wnt/β-catenin plays a key role in the signaling pathway of cancer stem cells, IRF2 may influence the stem cell phenotype by regulating β-catenin in hepatocellular carcinoma.

Others have shown that KRAS^G12D^-mediated repression of IRF2 subsequently enhanced the expression of CXCL3 that binds to CXCR2 on myeloid-derived suppressor cells (MDSCs) and increases the migration of MDSCs to the CRC microenvironment, which results in higher IRF2 expression and more sensitive response to anti-PD-1 therapy *in vivo* [9]. These studies demonstrate that IRF2 plays a dual role in immunotherapy. Our findings show that IRF2 contributes to the drug resistance of Lenvatinib in HCC.

Lenvatinib, used as the first-line treatment for HCC tumors, represses tumor cells by targeting FGF/FGFR, RET protooncogene, and cKIT. The functions of these targets depend on the β-catenin pathway. FGF10 and FGFR-mediated signaling play a crucial role in a β-catenin signaling-dependent manner in liver stem cells, hepatic tumor-initiating stem cells [20,21] and CRC cells [22]. Furthermore, studies have shown that RET binds to β-catenin, inducing tyrosine phosphorylation, which enhances the nuclear accumulation of β-catenin to increase the cell growth of papillary thyroid carcinoma (PTC) *in vitro* and *in vivo* [23,24]; The downregulation of *RET* induced by miR-449 inactivated β-catenin-mediated transcription, cell proliferation, and transformation of PTC [25]. Moreover, cKIT enhances β-catenin activity and transcription of β-catenin target genes related to angiogenesis to induce neovascularization [26]. In our results, we identified IRF2 as a new upstream regulator of β-catenin and mediates the resistance of Lenvatinib in human HCC. Lenvatinib treatment decreased HCC viability, and silencing endogenous IRF2 further augmented this inhibition. In addition, the resistance of Lenvatinib induced by overexpression of IRF2 can be partially rescued by inhibiting β-catenin with XAV-939. In addition, we found that lenvatinib treatment upregulated the expression of IRF2 and β-catenin in HepG2 and Huh7 cells in a dose- and time-dependent manner, but the specific mechanism is unclear. Lenvatinib treatment is a selection process of cancer cells, resulting in a stronger viability and tendency to develop malignancy, which promotes the expression of oncogene IRF2 and β-catenin. The possible potential mechanisms include the direct regulation of lenvatinib at the transcriptional level, translation level, and/or post-translational modification of IRF2 and β-catenin. Another possibility is indirect regulation through lenvatinib target signaling, including FGF/FGFR, RET and cKIT. Further research is needed to verify these potential mechanisms. Collectively, these findings underscore the important role of IRF2 and β-catenin in the signaling pathway of hepatocellular carcinoma.

We also analyzed the downstream effects of β-catenin in IRF2-overexpressed/deficient HCC. c-Myc is known as an oncogene and a target of β-catenin [27]. Target genes of c-Myc contribute to various tumor processes involved in metabolic reprogramming, cancer stem cell-like properties, and therapeutic resistance, and play a crucial role in tumorigenesis and tumor progression [28,29].

## Conclusion

We identified IRF2 overexpression as an important mechanism underlying the drug resistance of Lenvatinib in human HCC. The expression of β-catenin and IRF2 was positively correlated in HCC tumors. Silencing endogenous IRF2 downregulated the expression of β-catenin, while overexpressing IRF2 upregulated β-catenin. These findings identify an important function of IRF2 in mediating Lenvatinib resistance of HCC cells. Targeting IRF2 may be a potential strategy to improve the therapeutic effect of Lenvatinib on HCC.

## Funding information

This work was sponsored by grants from the NIH NIDDH HHSN276201200017C and P30DK120531 (DAG).

## Author contribution

Design: Yarong Guo, David. A. Geller; Methodology: Yarong Guo, Qiang Du, Yihe Yan; Clinical and animal data: Yarong Guo, Qiang Du; Data analysis: Yarong Guo, David. A. Geller, Jun Xu; Writing and reviewing manuscript: Yarong Guo, David. A. Geller; Technique support: Yarong Guo, Qiang Du, Yihe Yan; Study supervision: David. A. Geller.

## Declaration of Competing Interest

The authors declare no conflicts of interest.

## Appendix Supplementary materials

Image, application 1
